# Magnesium Replacement to Protect Cardiovascular and Kidney Damage? Lack of Prospective Clinical Trials

**DOI:** 10.3390/ijms19030664

**Published:** 2018-02-27

**Authors:** Juan R. Muñoz-Castañeda, María V. Pendón-Ruiz de Mier, Mariano Rodríguez, María E. Rodríguez-Ortiz

**Affiliations:** 1 Nephrology Service, Instituto Maimónides de Investigación Biomédica de Córdoba (IMIBIC), University Hospital Reina Sofía, University of Córdoba, 14004 Córdoba, Spain; juanr.munoz.exts@juntadeandalucia.es (J.R.M.-C.); mvictoriaprm@gmail.com (M.V.P.-R.d.M.); marien_rguez@hotmail.com (M.E.R.-O.); 2 Red de Investigación Renal (REDinREN), Instituto de Salud Carlos III, 28029 Madrid, Spain

**Keywords:** magnesium, chronic kidney disease, cardiovascular disease, vascular calcification, mortality

## Abstract

Patients with advanced chronic kidney disease exhibit an increase in cardiovascular mortality. Recent works have shown that low levels of magnesium are associated with increased cardiovascular and all-cause mortality in hemodialysis patients. Epidemiological studies suggest an influence of low levels of magnesium on the occurrence of cardiovascular disease, which is also observed in the normal population. Magnesium is involved in critical cellular events such as apoptosis and oxidative stress. It also participates in a number of enzymatic reactions. In animal models of uremia, dietary supplementation of magnesium reduces vascular calcifications and mortality; in vitro, an increase of magnesium concentration decreases osteogenic transdifferentiation of vascular smooth muscle cells. Therefore, it may be appropriate to evaluate whether magnesium replacement should be administered in an attempt to reduce vascular damage and mortality in the uremic population In the present manuscript, we will review the magnesium homeostasis, the involvement of magnesium in enzymatic reactions, apoptosis and oxidative stress and the clinical association between magnesium and cardiovascular disease in the general population and in the context of chronic kidney disease. We will also analyze the role of magnesium on kidney function. Finally, the experimental evidence of the beneficial effects of magnesium replacement in chronic kidney disease will be thoroughly described.

## 1. Magnesium: Metabolism and Physiology

Magnesium (Mg) is one of the most abundant cations in organisms [1], and it is involved in a number of physiological processes such as enzymatic reactions and membrane and structural functions [2].

In health, total Mg levels range between 0.7 and 1.4 mM. Johansson et al. compared the levels of ionized and total Mg, finding a weak correlation between both forms [3]. Bone is the main reservoir of Mg (60–65%), buffering changes in Mg level; tissue compartments, mainly skeletal muscle, represent approximately 35% of total Mg, whereas only 1–2% Mg is present in the extracellular fluid [1]. Serum Mg can be found in three different forms: (1) ionized, which mainly exerts biological actions (55–70%), (2) bound to proteins (20–30%) and (3) forming complexes with phosphate, citrate and bicarbonate (5–15%) [2,4]. Assessment of Mg levels is normally performed by measuring total serum Mg. However, this parameter may not reflect accurately the actual Mg availability (ionized Mg) due to the fact that it is influenced by factors such as pH or the presence of other ligands; this is of particular importance in the population with chronic kidney disease (CKD), in which advanced stage ionized Mg levels are affected by high serum phosphate and a high anion gap [5]. The vast majority of Mg is stored in bone, muscle and at the intracellular level, which may further impair a precise evaluation of Mg status [6].

**Figure 1:**
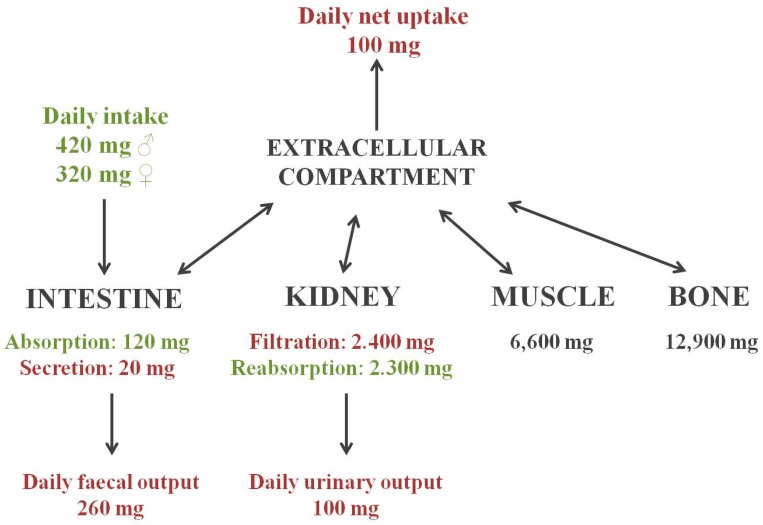
Overview of magnesium homeostasis.

According to the U.S. Institute of Medicine (Washington DC), daily Mg intake in men and women is estimated to be 420 and 320 mg/day, respectively. Approximately 50% of Mg is absorbed, although this proportion varies according to the dietary content of other elements such as protein or fiber [7]. Three different organs are responsible for Mg homeostasis: intestine, where absorption takes place; bone, responsible for storage; and kidneys, controlling Mg excretion. Intestinal Mg absorption occurs through two different paths: paracellular transport, a passive mechanism that represents 80–90% of intestinal uptake, and transcellular absorption, which involves the participation of the transient receptor potential channel melastatin members 6 and 7 (TRPM 6 and TRPM7) [8]. As mentioned, bone is essential for Mg storage, and it has been shown that dietary Mg influences bone metabolism [9]. Magnesium reabsorption takes place in the various parts of the nephron through different mechanisms: passive paracellular transport occurs in the proximal tubule and the thick ascending limb, where 10–25% and 70% of Mg is absorbed, respectively. Claudins are tight-junction proteins that determine the selectivity to small ions and neutral solutes, and most of them are expressed in the renal tubule [10]. Claudins 16 and 19 have relevant roles in the paracellular transport of Mg in the thick ascending limb, and mutations in their genes cause Mg wasting [11,12]. Furthermore, the TRPM6 channel enables the active transport of Mg predominantly in the distal convoluted tubule, where approximately 10% of Mg is reabsorbed [8]. A scheme of Mg homeostasis is depicted in Figure 1.

## 2. Magnesium and Enzyme Activity

Magnesium acts as a cofactor in reactions related to glycolysis [13], cell respiration [14,15] and the transport of cations across membranes [16]. Magnesium participates in enzymatic reactions [2,17] in several ways: binding to the ligand, binding to the active site of the enzyme, inducing a conformational change during the catalytic process, promoting the formation of multi-enzyme complexes or the combination of some of these. When forming complexes with ATP or GTP, Mg is the substrate for kinases B, ATPases or GTPases and cyclins. Furthermore, Mg is directly involved in the activation of enzymes such as phosphofructokinase, adenylate cyclase and Na^+^ and K^+^-ATPase [18]. In the context of mineral metabolism and its derangements in renal disease, many of these enzymes are key for the normal release of parathyroid hormone (PTH).

## 3. Magnesium and Apoptosis

Apoptosis is a mechanism of programmed cell death, necessary to eliminate damaged or unneeded cells, but it is also a physiological response under cellular stress. Magnesium itself has a prominent role in the onset of apoptosis. An increase in intracellular Mg is observed in cells undergoing apoptotic death, and this precedes DNA fragmentation; it has been hypothesized that mitochondria are the primary source of Mg, since treatment with an inhibitor of mitochondrial oxidative phosphorylation reduced the proportion of cells mobilizing Mg and the degree of DNA fragmentation, one of the hallmarks of apoptosis [19].

Divalent cations are essential for the normal functioning of the cell. A number of pieces of evidence points to the proapoptotic role of Mg deficiency. In rats, Malpuech-Brugère and collaborators reported an association between low Mg and early thymus involution that was accompanied by histological changes and an elevated apoptosis rate [20]. In addition, low Mg concentrations promote apoptosis in cultured rat hepatocytes, although Mg supplementation does not prevent the spontaneous apoptosis that normally occurs in this type of cell culture [21]. Similar observations have been reported in the vasculature. Li and collaborators found that the decline in Mg associated with the treatment with peroxynitrite triggered apoptosis in cultured vascular cells and was attenuated by the addition of Mg [22]. In vivo, Mg dietary deficiency is related to increased DNA fragmentation and caspase-3 activity in cardiac and vascular tissue; by contrast, Mg supplementation abolishes such effects [23]. According to the work by Feng et al., the scavenger receptor BI (SR BI) is involved in the mechanism underlying the apoptosis dependent on Mg deficiency [24].

## 4. Magnesium and Oxidative Stress

Multiple studies have linked Mg deficiency and negative cardiovascular outcomes (reviewed in [25,26,27]), and a considerable amount of evidence points to exacerbated oxidative stress as one of the mechanisms contributing to such deleterious effects.

Shivakumar and collaborators showed for the first time an increase in TBARS (thiobarbituric acid-reactive substances), an indicator of increased oxidative activity, in plasma and aorta from rats fed with a Mg-deficient diet, along with an increased activity of antioxidant enzymes [28]. The experimental administration of a Mg-deficient diet for 21 days produced an increase in lipid peroxidation at cardiovascular level, which was prevented with Mg supplementation [23]. Also in line with these findings, Mg administration prevented the cardiovascular increase in lipid peroxidation following heart injury in dogs [29]. The direct relationship between low Mg and oxidative damage was confirmed in vitro in aortic endothelial cells by Dickens et al., who also reported a concomitant and acute negative effect on cell viability [30]. In this regard, a possible involvement of low Mg-induced oxidative stress in processes related to endothelial dysfunction has been suggested [31]. Such effects may be supported by the involvement of reactive oxygen species in hypertension, endothelial dysfunction and vascular remodeling caused by chronic Mg deficiency [32].

Other tissues are also susceptible to suffer oxidative stress by Mg insufficiency. The skeletal muscle of rats receiving a Mg-deficient diet for 12 days exhibited higher production of free radicals that were accompanied by ultrastructural abnormalities [33]. A similar effect has also been reported in liver [34]. Furthermore, low Mg may produce changes in mineral homeostasis, and it is also associated with chronic hyperglycemia, which contributes to the increase in oxidative stress in diabetes type 2. In a case-control study, Araujo-Sampaio et al. found, among other mineral disturbances, a higher incidence of hypomagnesemia in spite of a significantly more elevated consumption of Mg, which might partially contribute to the marked increase in the formation of TBARS found in plasma and suggest an inadequate Mg handling in this pathology [35].

According to recent findings, the relationship between low Mg and oxidative stress is bidirectional, and oxidative stress may also exacerbate Mg deficiency. Kolisek and collaborators hypothesized that the upregulation of the protein PARK7/DJ-1, due to increased oxidative stress, may underlie the changes in Mg level through promoting the transcription of the Na/Mg exchanger SLC41A1 [36].

Taken together, all these pieces of evidence point out to both antiapoptotic and antioxidant beneficial effects of normalizing serum Mg concentration. Nevertheless, interventional studies in humans are needed to further explore the beneficial impact of Mg supplementation on apoptosis- and oxidative stress-related parameters.

## 5. Clinical Association between Magnesium and Cardiovascular Disease in the General Population

Magnesium has vasodilatory, anti-inflammatory, anti-ischemic and antiarrhythmic properties; thus, it is presumably a useful therapeutic agent in cardiovascular medicine. Several studies have established the role of Mg in the pathogenesis of cardiovascular disease (CVD) in the general population [37,38]. In the general population, hypomagnesemia is normally observed in diabetes, chronic gastrointestinal diseases, alcoholism and the use of certain drugs. In hospitalized patients, the prevalence of hypomagnesemia is estimated to be between 9% and 65% [39]. Epidemiology studies show that low levels of serum Mg may increase the risk of CVD [40,41]. Accordingly, several meta-analyses suggest that the intake of Mg is associated with a reduced incidence of CVD [42,43]. A summary of the main studies reported in this regard is shown in Table 1.

In a prospective study of Mediterranean individuals at high risk of CVD, an inverse association between dietary Mg intake and risk of mortality was found; however, no significant associations with cardiovascular events were observed [44]. In a prospective cohort of women, higher Mg intake and serum Mg were associated with a lower risk of fatal coronary heart disease (CHD) [45]. In another prospective cohort of older adults, plasma Mg concentration was inversely related to all-cause mortality risk, but not to dietary Mg intake. High plasma Mg was associated with a 29% lower risk of all-cause mortality [41]. Other prospective studies show that adults at high CVD risk who had the highest Mg intake were at a 37% lower risk of all-cause mortality [41,44].

A meta-analysis evaluating the association between Mg and the risk of cardiovascular events demonstrated that both dietary and serum Mg were inversely related to the risk of total CVD events [42]. Likewise, inverse associations between dietary Mg intake and the risk of stroke or ischemic heart disease were also demonstrated in other meta-analyses [43]. In the most recent meta-analysis about this issue, elevated intake of dietary Mg was associated with a reduced risk of stroke, heart failure, diabetes and all-cause mortality, but not with CHD or total CVD. In fact, it was associated with a 22% reduction in the risk of heart failure and a 7% reduction in the risk of stroke [40].

Consequently, the majority of recent studies support an inverse correlation between dietary Mg intake and serum Mg levels and the risk of CVD and mortality.

## 6. Clinical Association between Magnesium and Cardiovascular Disease in CKD

Cardiovascular disease is the leading cause of mortality in the CKD population [46]. In CKD, the reabsorption of Mg is progressively adapted as the glomerular filtration rate (GFR) decreases, in an attempt to maintain serum Mg within a physiological range. The incidence of hypomagnesemia was recently evaluated in a population of hemodialysis individuals, reporting 12% of hypomagnesemic patients [47]. Magnesium intake is the main reason for the Mg serum levels in hemodialysis patients [48]. Importantly, due to potassium dietary restriction, CKD patients are at risk of hypomagnesemia given that foods rich in this element such as green vegetables and nuts also contain elevated amounts of Mg [49].

Magnesium has been shown to impact cardiovascular health positively [38,50,51,52]. A large registry-based cohort study by Sakaguchi and collaborators revealed the role of Mg as a predictor of all-cause and cardiovascular mortality in end-stage renal disease; patients in the lowest sextile of plasma Mg showed higher mortality rates [53]. Hypomagnesemia is tightly linked to the development of diabetes [54,55], and diabetes represents an important risk factor associated with CVD in uremia. In a population of type 2 diabetic patients, low Mg has been shown to predict cardiovascular mortality [56].

Magnesium has been shown to be related to different aspects of CVD. Human studies concerning this issue are summarized in Table 2.

### 6.1. Vascular Calcification

Coronary artery calcification (CAC), as a measure of advanced atherosclerosis, is a predictor of CVD [57]. In the absence of CKD, Mg has been inversely related to CAC in two cross-sectional studies. Hruby et al. reported 22% lower CAC score per increment of 50 mg/day in Mg intake in patients free of CVD [58]. Similarly, in another study including 1276 patients with no symptoms of CVD, those patients in the highest quartile of serum Mg (2.20–2.29 mg /dL) had significantly lower odds of CAC than those in the lowest quartile (1.83–1.94 mg/dL), *p* = 0.016 [59]. To our knowledge, only one study has assessed so far the association between magnesium and CAC in the setting of CKD. In predialysis patients with a mean eGFR of 35.7 mL/min/1.73 m^2^, an inverse relationship was found between serum Mg and CAC density, but not area. This relationship was also observed after adjusting for malnutrition-inflammation-atherosclerosis- and mineral and bone disorder-related parameters [60]. The experimental design of a multicentric randomized double-blind placebo-controlled clinical trial assessing the impact of the administration of oral Mg has been recently published [61]. This study is intended to evaluate the effect of the administration of a daily dose of 30 mmol of elemental Mg in predialysis patients with an eGFR range of 15–45 mL/min/1.73 m^2^ on the prevention of the progression of CAC, and it is expected to shed light on the impact of the handling of serum Mg in the progression of vascular calcification (VC).

### 6.2. Intima-Media Thickness

Intima-media thickness (IMT) appears to be influenced by Mg. In an observational study, 36 CKD patients at Stage 5 (eGFR < 15 mL/min/1.73 m^2^) and 61 individuals with no CKD (eGFR > 60 mL/min/1.73 m^2^) were allocated into two groups according to plasma Mg level [62]. Both high and normal Mg levels were defined as 0.90–1.32 mmol/L and 0.62–0.89 mmol/L, respectively. IMT did not differ significantly according to Mg concentration in controls. However, normal Mg was associated with higher IMT in both carotid arteries when compared with high Mg. Interestingly, patients with 0.90–1.32 mmol/L Mg had pulse wave velocity values similar to those observed in patients with normal renal function. Results in line with these have been recently reported in a pediatric population [63]. The effect of oral Mg supplementation has also been tested in hemodialysis patients. After two months of administration of Mg citrate, patients exhibited an improvement in IMT in both left (*p* = 0.001) and right (*p* = 0.002) carotids, despite showing a similar index at baseline [64]. Likewise, the use of Mg oxide has yielded similar results; after six months of administration, patients had a decrease in IMT, even after adjustment by other factors capable of influencing the outcome, such as hyperlipidemia, hypertension or diabetes mellitus [65].

### 6.3. Pulse Pressure

Magnesium has been also shown to influence pulse pressure (PP) in patients with CKD Stages 2–4, defined in terms of the estimated GFR according to the formula derived from the Modification of Diet in Renal Disease Study (MDRD) [66]. Pulse pressure was calculated as the difference between systolic and diastolic blood pressure, and patients were allocated into two groups, with PP values lower and higher than 50 mmHg, respectively. Both eGFR (*p* < 0.001) and plasma Mg concentration (*p* = 0.0001) differed between both groups. Magnesium levels diminished according to the progression of CKD and were significantly associated with increased PP (OR = 0.550; 95% CI, 0.305–0.727, *p* = 0.016).

### 6.4. Heart Failure

Although not in the setting of renal disease, an association between low serum Mg levels and the incidence of heart failure has been assessed in a large cohort of the population included in the ARIC (Atherosclerosis Risk in Community) Study. For this purpose, patients with prevalent heart failure were excluded from the study. After stratifying according to Mg levels (mean serum Mg was 1.63 ± 0.16 mEq/L), patients in the lowest category showed higher risk of incident heart failure (HR = 2.58; 95% CI, 2.23–2.97); this relationship remained significant after subsequent adjustments [67]. It remains to be clarified whether this association is also present when kidney function is diminished.

### 6.5. Dyslipidemia

Dyslipidemia, also a risk factor for CVD, is exhibited by CKD patients. Studies connecting Mg and dyslipidemia have yielded inconsistent results. On the one hand, Robles and collaborators observed a linear correlation between Mg and total cholesterol (r = 0.55, *p* < 0.001), LDL-cholesterol (r = 0.52, *p* < 0.01), VLDL-cholesterol (r = 0.49, *p* < 0.001) and ApoE (r = 0.52, *p* < 0.01) [68]. Ansari et al. reported a positive correlation between Mg and serum lipoprotein A (r = 0.40, *p* < 0.007), serum HDL (r = 0.31, *p* < 0.01) and serum triglycerides (r = 0.35, *p* < 0.005) in end-stage renal disease [69]. Baradaran and collaborators also found positive associations between Mg and lipoprotein A (r = 0.65, *p* < 0.05) and triglycerides (r = 0.32, *p* < 0.05), but not with HDL cholesterol [70]. By contrast, Dey et al. found a relationship between hypomagnesemia and dyslipidemia in patients in CKD Stages 2–5; in particular, Mg levels were found to be significantly associated with total, HDL, LDL and non-HDL cholesterol; in addition, all these parameters correlated with CKD severity [71].

### 6.6. Inflammation

The anti-inflammatory properties of Mg have been repeatedly reported. In experimental studies, inflammatory markers are elevated following dietary Mg deprivation [72]; if prolonged, the pro-inflammatory state induced by low Mg might lead to impaired organ function [73]. A relationship between low Mg status and inflammation in CKD patients has also been suggested [74,75,76]. In vitro experimental approaches may help elucidate the mechanisms underlying this effect; in endothelial cultured cells, Mg has been shown to activate NFκB and promote the secretion of inflammatory cytokines [77], therefore inducing a proatherogenic and proinflammatory environment [78].

Taken together, these findings suggest a direct relationship between Mg and various parameters related to cardiovascular health. Nevertheless, interventional studies are desirable for a better understanding of the impact of the restoration of Mg levels on the cardiovascular health in the context of renal disease. 

## 7. Hypermagnesemia and Mortality

Hypermagnesemia is uncommon in the normal population given the ability of the kidneys to remove the excess of Mg. The presence of hypermagnesemia has been associated with higher mortality in hospitalized [79], emergency [80], intensive care [81,82] and cardiac [83,84] patients.

In the context of renal disease, two different studies have evaluated the mortality risk in hemodialysis patients with low and high Mg levels, finding better survival rates in the latter [85,86]. Nevertheless, the results from different studies are not totally uniform; in a recent study, the authors did not report an additional risk for mortality in patients with high Mg levels [87].

## 8. Magnesium and Renoprotection

A number of pieces of evidence points to the role of Mg in the prevention or reversion of the renal damage subsequent to the therapeutic administration of nephrotoxic drugs of use in clinical practice. In spontaneously hypertensive rats, Mg supplementation alone or in combination with potassium has been shown to protect against the nephrotoxicity induced by cyclosporine [88]. On the other hand, the use of cisplatin, a chemotherapy agent, is associated with a high risk of acute kidney injury (AKI) and eventually CKD [89]. Administration of cisplatin causes hypomagnesemia in treated patients [90], which in turn is one of the mechanisms participating in the nephrotoxicity of cisplatin. In vivo studies have demonstrated that acute markers of kidney damage (blood urea nitrogen, creatinine and tubule injury signs) improved after Mg replacement when co-administered; interestingly, Mg does not seem to affect the antitumoral efficacy of cisplatin [91,92]. The renoprotective effect of Mg has also been evidenced by Saito and collaborators in cisplatin-treated cancer patients. Premedication with Mg was associated with lower nephrotoxicity measured in terms of changes in serum creatinine and creatinine clearance [93]. The same authors investigated the mechanism underlying this effect, finding that Mg prevents the downregulation of renal TRPM6 while inhibiting the organic cation transporter 2 (Oct2); both actions lead to prevention of both Mg wasting and accumulation of platinum [94]. In an animal model of diabetes using multiple low doses of Streptozocin to normal rats, parameters of kidney damage (elevations in blood urea nitrogen and markers of oxidative stress) were reversed by Mg treatment [95].

All these pieces of evidence entail an active role of Mg in preserving renal function, following the administration of certain drugs or even in the context of diabetic nephropathy. However, additional interventional studies would be desirable to confirm these observations in clinical practice, as well as to gain understanding of the mechanisms underlying this protective effect.

## 9. Magnesium and CKD Progression

Low Mg levels have been associated with a high risk of incident CKD or end-stage renal disease in a population of individuals with eGFR higher than 60 mL/min/1.73 m^2^ [96]. When CKD is established, hypomagnesemia predicts loss of kidney function [97]. In diabetic nephropathy, low plasma Mg has been associated with a faster decline in renal function [98] and with progression to end-stage renal disease [99]. In addition, levels of Mg determine the progression of CKD induced by hyperphosphatemia [100], which is a well-known risk factor for the loss of renal function [101]. We have also shown in an experimental model of uremia that dietary Mg halts the progression of renal disease in an effect that is mediated by a reduction in serum phosphorus levels [102]. However, other effects independent of phosphorus lowering such as inflammation, oxidative stress, dyslipidemia and hyperparathyroidism, all of them implicated in the progression of CKD, cannot be ruled out.

## 10. Experimental Evidence of the Beneficial Effects of Magnesium Replacement in CKD

**Figure 2:**
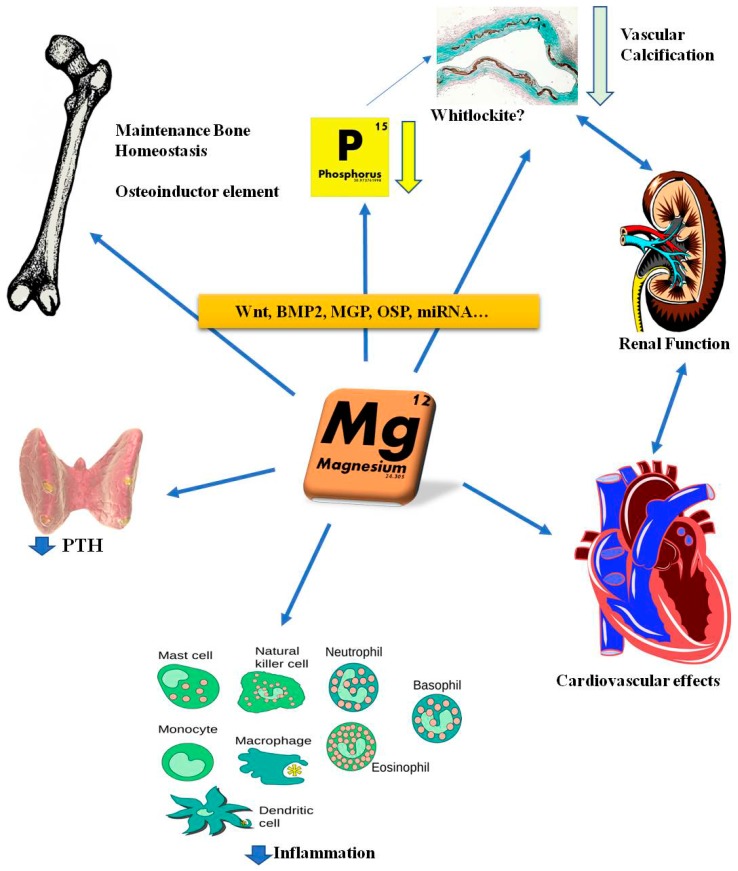
The pleiotropic effects of magnesium have been reported in several pathological conditions, showing beneficial effects at bone, renal and cardiovascular levels.

Experimental evidence suggests a beneficial effect of Mg replacement to manage the complications associated with the progression of CKD. Such evidence (summarized in Figure 2) has been supported by both in vivo and in vitro studies and will be described extensively below.

### 10.1. Serum Magnesium and PTH Levels

We have previously demonstrated that under conditions of moderate hypocalcemia, Mg through the activation of the calcium-sensing receptor reduces PTH, and this is accompanied by the upregulation of both the calcium-sensing receptor and vitamin D receptor [103]. Remarkably, this finding has been evidenced in the clinical setting by Sakaguchi et al. [104]; the presence of low or high calcium levels minimized the suppressive effect of Mg on PTH in patients undergoing hemodialysis. Matsuzaki et al. [105] observed in vivo that the dietary supplementation of Mg produces a decrease in PTH. Recently, Zhang et al. [106] identified residues in the extracellular domain of the calcium-sensing receptor key for Mg binding. This finding suggests a direct intervention of Mg on the decrease in PTH. Therefore, in the context of CKD, Mg replacement may help control PTH levels. In fact, the work by Navarro et al. [107] showed the inverse association between serum Mg concentration and PTH levels in dialysis patients.

### 10.2. Effect on Vascular Calcification

In the context of CKD, Mg supplementation is of particular interest given its ability to bind phosphorus and control hyperphosphatemia. In this regard, calcium acetate/magnesium carbonate has been proven to be effective in controlling serum phosphorus in dialysis patients [108].

Numerous experimental studies have shown that moderately high levels of Mg are instrumental in decreasing vascular calcifications (VC) [109,110]. Our group has shown recently that in rats with renal failure, dietary Mg supplementation was not only able to prevent the occurrence of VC, but also to reverse it. Magnesium reduces VC through the decrease in serum phosphate levels and by other mechanisms independent of phosphorus, resulting in improved survival [102]. Prevention of VC by Mg could be explained by two mechanisms: the action of Mg in preventing the formation of hydroxyapatite (passive process) and a second active mechanism, avoiding the transdifferentiation of vascular smooth muscle cells into an osteogenic phenotype. Both processes are supported by previous findings; next, they will be reviewed, and other potential mechanisms will be also proposed.

#### 10.2.1. Passive effect of Magnesium Supplementation

In the context of CKD, Mg exerts a binding effect that allows the reduction in serum phosphorus and VC. According to several publications, Mg exhibits a greater phosphorus binding capacity than calcium [111] when displacing the formation of hydroxyapatite towards whitlockite [112]. However, it has been also suggested that the beneficial effect of Mg is not solely due to a defect in the formation, composition and structure of hydroxyapatite crystals, but it also involves an active cellular effect [113]. It is interesting to note that hydroxyapatite and whitlockite deposits have been equally observed in the aorta of CKD patients, suggesting that there must be other additional mechanisms participating in the beneficial cardiovascular effects of Mg. Schutter and collaborators suggested that the formation of whitlockite in experimental models of VC is associated with excessive doses of vitamin D [114].

#### 10.2.2. Active Effect of Magnesium Supplementation

Our research group has demonstrated that the addition of Mg prevents and reverses phosphorus-induced calcification of human aortic vascular muscle cells. This is not merely a passive effect, but it depends on an active Mg transport across the cell membrane through the TRPM7 channel. Inhibition of TRPM7 with 2-aminoethoxy-diphenylborate (2-APB) or silencing the TRPM7 gene prevented the anti-calcifying effect of Mg [115]. We also showed that the activation of the Wnt/ß-catenin pathway, which mediates high phosphorus-induced calcification, can be prevented by moderate amounts of Mg that also increase the levels of Dkk-1, an endogenous inhibitor of the Wnt/ß-catenin pathway.

Other in vitro studies hypothesize the contribution of other mechanisms to the inhibition of VC produced by Mg. Thus, Mg supplementation is also associated with changes in the expression of microRNAs related to calcification [116]. miR-30b, miR-133a and miR-143 are downregulated in phosphorus-induced calcification, whereas the addition of Mg restored (miR-30b) or increased (miR-133a, miR-143) their expression. Interestingly, Mg in vitro also avoids the decrease in the expression of molecules such as MGP, osteopontin or BMP-7 [117,118], all of them calcification inhibitors.

### 10.3. Other Effects of Magnesium

In addition to these passive and active effects on phosphorus control and calcification, there is another set of actions key for Mg to develop its beneficial effects. We have shown that 14 days of Mg supplementation reduces serum creatinine in an experimental model of calcification, although these effects may be subordinated to the reductions in serum phosphorus, VC and PTH control [102]. In this model, the intraperitoneal administration of Mg resulted in a lesser degree of aortic calcification, despite no changes in serum phosphorus, which suggests an independent effect of Mg beyond its phosphorus binding action. On the other hand, human vein umbilical cells (HUVEC) treated with TNF alpha exhibit higher levels of BMP2 and p65, pro-calcificant and pro-inflammatory proteins respectively, which were reduced with the addition of Mg [102]. In this regard, several pieces of evidence point out that Mg deficiency promotes the generation of reactive species of oxygen and oxidative stress in endothelium [31,119]. Other works also support this beneficial action of Mg at the endothelial level in the context of atherosclerosis [120,121]. These findings, along with additional anti-inflammatory [122] and anti-apoptotic [123] actions, help to understand the complex mechanisms whereby Mg acts at the cardiovascular level.

### 10.4. Magnesium and Bone

Information on the bone effects of Mg is not uniform. It is recognized that a precise control of Mg homeostasis is essential for bone health [124]. Mg deficiency affects crystal formation, which contributes to osteoporosis, PTH activity and promotes low-grade inflammation. By contrast, little is known about the pathogenesis of the mineralization defects occurring in the setting of hypermagnesemia. It has been also demonstrated that Mg enhances osteogenesis of mesenchymal stem cells [125]. Within the context of biomaterials science, there is a growing interest in fixing in ceramic biomaterials or scaffolds made of alloys of Mg to improve osteogenesis and the osteointegration of the prosthesis used in traumatology surgery [126]. The dual effect of Mg supplementation in human osteoblasts has been recently reported, finding that concentrations higher than 4 mM of Mg decrease osteogenesis, while moderate concentrations of Mg increase mineralization [127]. These results are in line with other in vivo observations recently published [102].

In summary, experimental evidence suggests that oral Mg supplementation reduces serum phosphorus and has a direct protective effect against VC by inhibiting pro-calcificant pathways and reducing apoptotic and inflammatory cellular response.

## 11. Risk of Magnesium Overdose

Magnesium intoxication is not frequent. It is important to differentiate the risk of hypermagnesemia due to oral Mg supplementation from intravenous Mg treatment.

There are some isolated iatrogenic parenteral overdoses of Mg reported in the literature that have resulted in cardiopulmonary arrest. Manifestations of hypermagnesemia are dose-related. Minor side effects of parenteral Mg include flushing, warmth, nausea, headache and lightheadedness. Major, life-threatening effects involve the cardiovascular and neuromuscular systems. Hypermagnesemia is associated with absent deep tendon reflexes, apnea, coma, complete heart block and asystole, the latter with Mg concentrations above 8 mM [128].

Magnesium supplementation is well tolerated, although it may cause gastrointestinal symptoms including diarrhea, nausea and vomiting [129]. In a current clinical trial concerning oral magnesium supplementation in 34 patients with CKD Stages 3–4 during eight weeks, intracellular Mg was not increased, and there were no incidences of symptomatic hypermagnesemia. Magnesium supplementation was safe and well tolerated with no adverse events related to magnesium treatment [130].

Furthermore, in terms of Mg toxicity, pharmacological interactions should be taken into account. Concomitant oral intake of Mg may influence the absorption of aminoglycosides, bisphosphonates, calcium channel blockers, fluoroquinolones, skeletal muscle relaxants and tetracyclines.

## 12. Are We Ready for Magnesium Supplements in CKD?

Being one of the most abundant elements in the organism, Mg is essential for the normal development of a wide number of cellular functions. Magnesium deficiency is associated with deleterious effects, both at the cellular and systemic level. At the cellular level, low Mg is related to the occurrence of apoptosis and increased oxidative stress. At the systemic level, it is important to emphasize the association established between decreased Mg levels and CVD. Such a relationship has been repeatedly reported in the general population, but also in the context of CKD in terms of VC, IMT, PP and dyslipidemia, which is also related to the appearance of cardiovascular events. Taken together, experimental evidence strongly suggests a beneficial effect of the restoration of Mg levels when it comes to cardiovascular health. However, the vast majority of observations have been originated in experimental or observational studies. Therefore, there is an unmet need for prospective clinical trials that help elucidate the impact of Mg supplements on the cardiovascular health of CKD patients.

## Figures and Tables

**Table 1:** Summary of studies evaluating the effect of magnesium on cardiovascular-related outcomes in the general population.

Reference	Study Type	Clinical Setting	No. of Subjects	Outcome	Conclusion
Fang et al. [40]	Meta-analysis of prospective studies	General population	>1,000,000	CVD (coronary heart disease, ischemic heart disease, stroke) and all-cause mortality	Increasing dietary Mg is associated with a reduced risk of stroke and heart failure, but not with total CVD, and all-cause mortality.
Huang et al. [41]	Observational	Elderly	1400	All-cause and cause-specific mortality	Low plasma Mg levels increase all-cause mortality.
Qu et al. [42]	Meta-analysis of prospective studies	General population	532,979	CVD	Inverse association between dietary Mg intake and CVD risk.
Del Globbo et al. [43]	Meta-analysis of prospective studies	General population	313,041	Incidence of CVD, including IHD	Plasma and dietary Mg are inversely associated with CVD risk.
Guasch-Ferré et al. [44]	Prospective	Individuals at high risk of CVD	7216	CVD and all-cause mortality	Mg intake is associated with a lower mortality risk in this population, but not with CV events.
Chiuve et al. [45]	Prospective	Women free of disease	86,323	CHD	Dietary Mg intake was inversely associated with fatal CHD.

CVD: cardiovascular disease; Mg: magnesium; IHD: ischemic heart disease; CV: cardiovascular; CHD: coronary heart disease.

**Table 2:** Summary of studies evaluating the effect of magnesium on cardiovascular-related outcomes in CKD patients.

Reference	Study Type	Clinical Setting	No. of Subjects	Outcome	Conclusion
Sakaguchi et al. [53]	Observational	Hemodialysis	142,555	Cardiovascular and non-cardiovascular mortality	Hypomagnesemia predicts cardiovascular and non-cardiovascular mortality.
Sakaguchi et al. [60]	Observational	Pre-dialysis	109	Density of CAC	CAC is inversely associated with serum Mg levels.
Bressendorf et al. [61]	Interventional	Pre-dialysis	250	Progression of CAC	Ongoing study.
Salem et al. [62]	Observational	Dialysis	36	IMT PWV	In CKD, Mg levels were inversely associated with the IMT of carotids and the PWV.
Zaher et al. [63]	Observational	Hemodialysis	25	IMT	Mg correlates inversely with IMT in pediatric CKD.
Turgut et al. [64]	Interventional	Hemodialysis	47	IMT	Carotid IMT improved following administration of Mg citrate.
Mortazavi et al. [65]	Interventional	Hemodialysis	54	IMT, FMD, CRP	Mg may be involved in the decrease in IMT in treated patients.
Fragoso et al. [66]	Observational	Pre-dialysis	80	PP	Low Mg levels are independently associated with higher PP.
Robles et al. [68]	Observational	Hemodialysis	25	Dyslipidemia	Mg is positively associated with total cholesterol, LDL-C, VLDL-C and ApoB.
Ansari et al. [69]	Observational	Hemodialysis	50	Dyslipidemia	Mg is directly associated with LP-A, HDL-C, and TG.
Baradaran et al. [70]	Observational	Hemodialysis	36	Dyslipidemia	Positive correlation between Mg and LP-A and TG.
Dey et al. [71]	Observational	Pre-dialysis	90	Dyslipidemia	Significant association between Mg, total cholesterol, HDL-C, LDL-C and non-HDL-C.

CAC: coronary artery calcification; Mg: magnesium; IMT: intima-media thickness; PWV: pulse wave velocity; FMD: brachial artery flow-mediated dilatation; CRP: C-reactive protein; PP: pulse pressure; LDL-C: LDL-cholesterol; VLDL-C: VLDL-cholesterol; ApoB: apolipoprotein B; LP-A: lipoprotein A; HDL-C: HDL-cholesterol; TG: triglycerides.
